# Rutin Inhibits Cardiac Apoptosis and Prevents Sepsis-Induced Cardiomyopathy

**DOI:** 10.3389/fphys.2022.834077

**Published:** 2022-04-14

**Authors:** Xiang-Long Meng, Mu-Ming Yu, Yan-Cun Liu, Yu-Lei Gao, Xin-Sen Chen, Song-Tao Shou, Yan-Fen Chai

**Affiliations:** Department of Emergency Medicine, Tianjin Medical University General Hospital, Tianjin, China

**Keywords:** sepsis, sepsis-induced cardiomyopathy (SIC), rutin, apoptosis, mitochondrial

## Abstract

Rutin is a flavanol-type polyphenol that consists of flavanol quercetin and the disaccharide rutinose, which has been reported to exert various biological effects such as antioxidant and anti-inflammatory activities. It is not clear whether rutin has a protective effect on sepsis-induced cardiomyopathy (SIC). In this study, we used male C57BL/6 mice and cecal ligation and puncture (CLP) surgery to establish the model of SIC. Rutin was precautionarily treated (50, 100, 200 mg/kg per day, 7 days) before CLP. The results showed that rutin pretreatment (100, 200 mg/kg per day, 7 days) reduced the mortality of murine sepsis. We chose the 100 mg/kg dose for further studies. Mice were pretreatment with rutin (100 mg/kg per day, 7 days) before subjected to CLP, and myocardial tissue and blood samples were collected 24 h after CLP. Serum levels of tumor necrosis factor-α (TNF-α), interleukin-6 (IL-6), and cTNT decreased, while interleukin-10 (IL-10) increased with rutin pretreatment. The cardiomyocytes apoptosis and mitochondrial dysfunction were also alleviated with rutin pretreatment. In conclusion, this study confirmed the efficacy of rutin-enriched diet in the prophylaxis of cardiac apoptosis and cardiac injury induced by CLP in mouse model. It provides a potential new approach on SIC prophylaxis in sepsis.

## Introduction

Sepsis is a dysregulated host response to infection that can eventually lead to multiple organ dysfunction syndrome (MODS) and is one of the most common causes of death among hospitalized patients (Evans et al., 2021). In North America, the 30-days mortality of septic shock is as high as 33.7% (Bauer et al., 2020). Sepsis-induced myocardiopathy (SIC) is a common and serious complication of sepsis. Compared to patients with sepsis without cardiac dysfunction, the mortality rate of SIC patients is significantly higher. Therefore, it is of great importance to clarify the pathogenesis of SIC for the future prevention and treatment of sepsis (Wang et al., 2019; Lin et al., 2020).

The heart, as the pump organ, plays a key role in the pathophysiology of septic shock. Cardiomyocyte apoptosis is the main mechanism of SIC. The stunned myocardium may also be involved in the pathogenesis of SIC. Revitalization of the failing myocytes may allow recovery of systolic ventricular function in SIC (Narula et al., 2001; Haider et al., 2002). Mitochondria, as the energy supplier of cardiomyocytes, contributes to the pathophysiology that underlies myocardial dysfunction in sepsis (Mantzarlis et al., 2017). Sepsis causes mitochondrial damage through numerous mechanisms (Santulli et al., 2015; Bugger and Pfeil, 2020). Damaged mitochondria promote cardiomyocyte apoptosis, which in turn aggravates septic cardiomyopathy. Therefore, interventions for mitochondrial damage and cardiomyocyte apoptosis may have benefits for SIC, which is worthy of further research.

Rutin, a natural flavonol glycoside, also referred to as vitamin P, extensively exists in buckwheat, cranberries, mulberry, and citrus fruits. Rutin has a wide range of beneficial health properties in multiple organs owing to its various pharmacological effects. Previous research has shown that rutin can reduce the level of oxidative stress *via* ROS-scavenging mechanisms and protect neurons against severe oxidative stress and attack by ROS, promising through neuroprotective properties therapy cognitive dysfunction in Alzheimer’s disease (AD) (Simunkova et al., 2019). Michele et al. show that limited ROS products can ameliorate mitochondrial function, protecting against cardiotoxicity caused by the anticancer drug (Russo et al., 2019). It also has been demonstrated that rutin has the properties of anti-inflammatory, antiallergenic, antiviral, and anticarcinogenic (Ganeshpurkar and Saluja, 2017a). However, whether rutin will have a protective effect on SIC is unexplored.

## Materials and Methods

### Rutin

**FIGURE 1:**
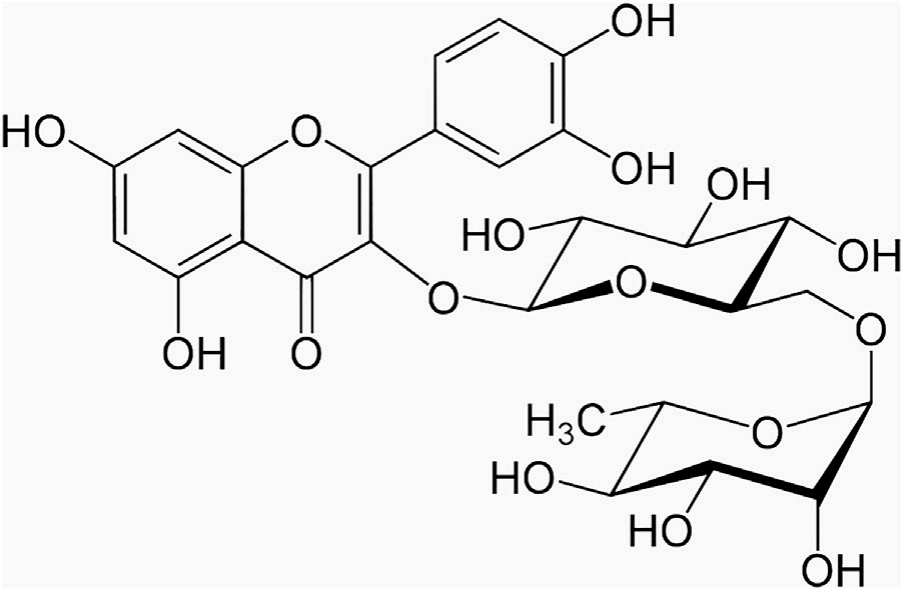
Chemical structure of flavonoid rutin.

Rutin (Figure 1 purity > 98%) was purchased from MCE Biotechnology Co., Ltd. (United States, #153184). 0.5% sodium carboxymethyl cellulose (NaCMC) was purchased from Sigma Biotechnology Co., Ltd. (United States, #419273). Rutin was dissolved in 0.5% NaCMC and was given to mice by gavage.

### Animals

Male C57BL/6 mice (23 ± 2 g, 8–10 weeks old) were obtained from Beijing WTLH Laboratory Animal Technology Co., Ltd. (Beijing, China). All mice were adapted for 1 week (temperature: 23 ± 1°C, humidity: 45%–50%) and kept in a 12 h/12 h light/dark cycle. All animals were raised with standard water and food. The CLP surgery was performed as follows. Surgery was performed under anesthesia by isoflurane (3%) delivered in oxygen at a rate of 1 L/min in an anesthetic chamber *via* a face mask. Buprenorphine (0.05 mg/kg) was administered ip 5 min before surgery to obtain adequate analgesia. The cecum was exposed by a 1 to 2 cm midline incision in the anterior abdomen, subjected to ligation of the distal half of the cecum, and punctured once with a 22 G needle in the ligated segment. The cecum was then repositioned, 1 ml of sterile saline (pyrogen-free 0.9% NaCl) was subcutaneously administered, and the incision was closed with 9 mm steel wound clips.

Our experiments were carried out in strict accordance with international ethical guidelines and the National Institutes of Health Guide on the care and use of laboratory animals. Animals were checked daily for signs of distress and endpoints. Specific criteria used to determine when the animals should be euthanized were in accordance with Remick lab report (Nemzek et al., 2004). All protocols were approved by the Institutional Animal Care and Use Committee of Tianjin Medical University. All animal studies followed the ARRIVE guidelines 2.0 (Percie du Sert et al., 2020).

### Comparison of Survival Rates in Different Groups

Male C57BL/6 mice received rutin (50, 100, 200 mg/kg per day) dissolved in NaCMC or NaCMC only for 7 days before being subjected to CLP-induced lethal sepsis. Animals were checked daily and recorded for up to 7 days. The survival rate was analyzed by the log-rank test in GraphPad Prism software and presented as Kaplan-Meier curves.

### Experimental Protocols

**FIGURE 2:**
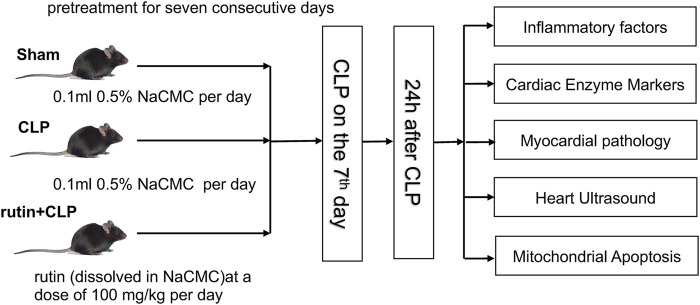
The animal handling process in this study.

Eighteen mice were randomly divided into a sham group (*n* = 6), CLP group (*n* = 6), and rutin+CLP group (*n* = 6). Sham group and CLP group received 0.5% NaCMC for seven consecutive days prior to surgery, while the rutin + CLP group received rutin (100 mg/kg) dissolved in 0.5% NaCMC for seven consecutive days prior to CLP (Figure 2). Cardiac function was determined by echocardiography 24 h after surgery. Immediately after this, the mice were sacrificed, and blood samples and heart tissues were retained for further investigation (three independent experiments were performed). The design of our study adheres to most of the points described in the Minimum Quality Threshold in Pre-Clinical Sepsis Studies (MQTiPSS) Consensus Recommendations (Osuchowski et al., 2018).

### ELISA Analysis

The level of TNF-α, IL-6, IL-10, and cTnT in plasma was determined by enzyme-linked immunosorbent assay (ELISA) kits according to the manufacturer’s instructions. The absorbance of each group was measured by a microplate reader at the wavelength of 450 nm. TNF-α, IL-6, and cTnT ELISA kits were purchased from Senbega Biotechnology Co., Ltd. (Nanjing, China), IL-10 ELISA kits were purchased from Beijing Dakewe Biotechnology Co., Ltd. (Beijing, China).

### Hematoxylin-Eosin (H&E) Staining

Heart tissues isolated from mice were completely fixed with 4% formaldehyde, then dehydrated with a gradient ethanol solution, soaked in wax, and embedded in paraffin in blocks. They were cut into 5 μm slices, and then deparaffinized and hydrated. The sections were stained with hematoxylin-eosin (H&E). The degree of cardiomyopathy was evaluated according to the degree of myocardial cell damage and the percentage of loss of myocardial fiber in this section. 0=no lesions, 1=myocardial damage <25%, 2=myocardial damage 25%–50%, 3=myocardial damage 50%–75%, 4=myocardial damage >75%.

### Mice Heart Ultrasound Echocardiography

The mice were anesthetized and placed on a heating pad to maintain their temperature. Measured echocardiographic indicators (VisualSonics, Canada) included the following: left ventricular end-diastolic volume (LVEDV), left ventricular end-systolic volume (LVESV), EF was calculated using the following formula: EF (%) = (LVEDV-LVESV)/LVEDV × 100%. At the same time, left ventricular fractional shortening (FS) was calculated, and all echocardiograms were performed by the same trained investigator.

### TUNEL Staining of the Mouse Heart

A commercial kit that uses fluorescein-dUTP to label DNA fragmentation sites (Servicebio, G1501) was used to detect cardiomyocyte apoptosis. Cut the myocardial tissue fixed with 4% paraformaldehyde into 4 μm slices. Heart sections were deparaffinized, rehydrated, and equilibrated in Tris buffered saline (TBS). Proteinase K (2 mg/ml) diluted with PBS (final concentration 20 μg/ml, pH 7.4–8.0) was then applied to the samples and incubated for 20 min at room temperature. The FITC-12-dUTP labeling mix was thawed and mixed with the buffer solution. The glass slide was immersed in the staining jar containing the DAPI solution in the dark and kept for 15 min at room temperature. Images were taken with a fluorescence microscope (Olympus, Japan), TUNEL-positive nuclei (fragmented DNA) were observed in green, while the nuclei locations (DAPI) were in blue.

### Western Blot Analysis

Murine heart tissue was homogenized in homogenization buffer (20 mM HEPES, pH 7.9, 1 mM MgCl_2_, 0.5 mM EDTA, 1% Nonidet P-40, 1 mM EGTA, 1 mM DTT, 0.5 mM PMSF, 1 μl/ml PIC) and centrifuged by 18,800 g for 40 min at 4°C. The supernatant was transferred to a fresh tube, and protein concentrations were determined using a BCA protein kit. Using 10% SDS-PAGE, separate the proteins (Solarbio, China), then transfer them to a polyvinylidene fluoride (PVDF) membrane (Millipore, United States). The membrane was blocked with TBST that contained 5% non-fat milk for 1 h at room temperature, then incubated with primary antibodies overnight at 4°C. Antibodies include Bcl-2 (Cell Signaling Technology, #3498; 1:1000), Bax (Cell Signaling Technology, #14796; 1:1000), Caspase-9 (Cell Signaling Technology, #9508; 1:1000), β-actin (Cell Signaling Technology, #3700; 1:1000). After three washing cycles, the membrane was incubated for 30 min with the HRP-conjugated secondary antibody. Protein bands were detected with an enhanced chemiluminescent (ECL) detection system. Finally, the membrane was exposed with a gel imaging system (Bio-Rad, United States). The immunoreactive bands were analyzed with ImageJ software.

### Transmission Electron Microscopy (TEM)

After obtaining fresh myocardial tissue samples from mice, they were quickly cut into small pieces on ice and fixed in 2% glutaraldehyde precooled for 2 days, tissues were placed in PBS buffer for 6 h and fixed with 1% osmium acid fixative for 3 h, then gradient ethanol dehydration and embedding with epoxy resin at room temperature. The samples were cut with an ultrathin microtome, then stained with 3% uranyl acetate lead citrate, observed, and photographed under a transmission electron microscope (HT7700, Japan). Then randomly select 5–8 fields, observe the characteristics of the mitochondrial structure, numbers, and calculate the mean mitochondrial area using ImageJ software.

### Statistical Analysis

Statistical analyzes were performed using Prism 9.0 (GraphPad Software, Inc., San Diego, CA). Data were expressed as mean ± SEM. A one-way ANOVA followed by the Bonferroni test was used for multiple comparisons. Statistical significance was established at a *p* value less than 0.05. Survival is analyzed using the log-rank test in GraphPad Prism software and presented as Kaplan-Meier curves.

## Results

### Rutin Improves the Survival Rate of CLP-Induced Septic Mice

**FIGURE 3:**
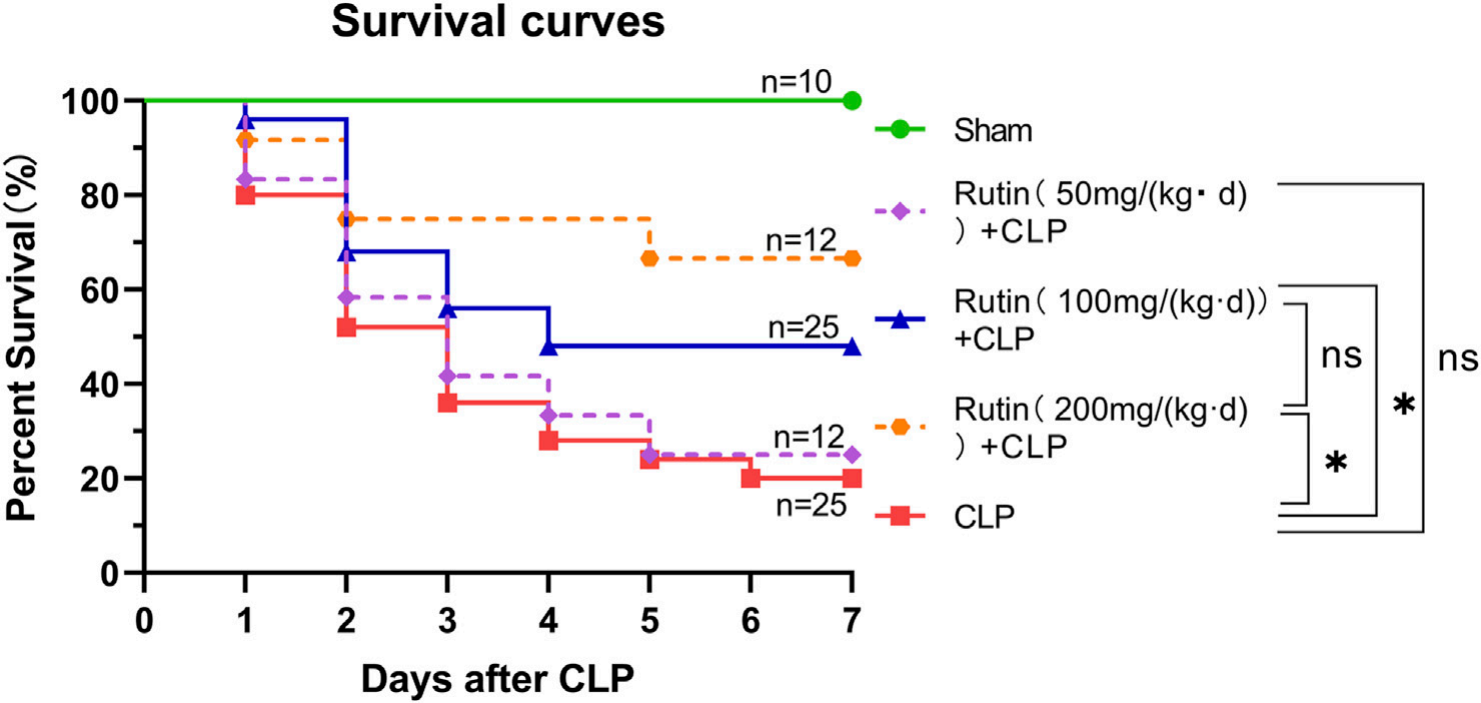
Rutin improved the survival rate of septic mice induced by CLP. Male C57BL/6 mice were orally given rutin (50, 100, 200 mg/kg per day) dissolved in NaCMC or NaCMC only for 7 days before being subjected to lethal sepsis induced by CLP. The survival rate was analyzed by the log-rank test in GraphPad Prism software and presented as Kaplan-Meier curves. * *p* < 0.05.

Male C57BL/6 mice were orally given rutin (50, 100, 200 mg/kg per day) dissolved in NaCMC or NaCMC only for 7 days before being subjected to lethal sepsis induced by CLP. There was no significant difference between the 50 mg/kg and the non-intervention group. Compared to the nonintervention group, doses of 100 mg/kg and 200 mg/kg significantly improved the survival rate of sepsis mice. There is no difference in survival between the 100 and 200 mg intervention groups (Figure 3). This result indicated that pretreatment with rutin (100 and 200 mg/kg per day) could protect mice against CLP-induced lethality. We chose the 100 mg/kg dose for further studies.

### Rutin Alleviates Inflammation and Decreases Myocardial Injury in Mouse Sepsis Model Induced by CLP

**FIGURE 4:**
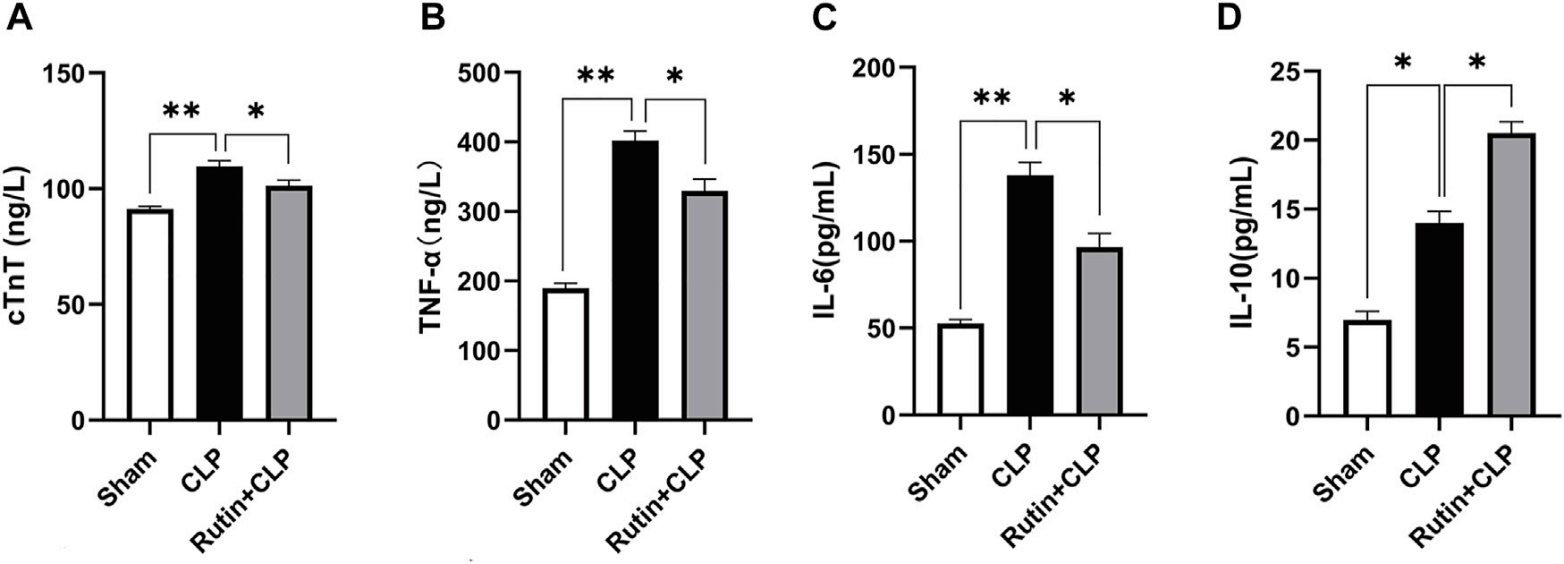
Rutin alleviates inflammation and decreases myocardial injury in the mouse sepsis model induced by CLP. Serum levels of cTnT **(A)**, TNF-α **(B)**, IL-6 **(C)**, and IL-10 **(D)** were measured using ELISA after 24 h of CLP. Data represented the mean ± SEM of independent experiment in triplicate (*n* = 6 per group). Statistical significance (*p < 0.05) was analyzed using one-way ANOVA.

Male C57BL/6 mice were precautionarily treated with rutin (100 mg/kg) for 7 days, then subjected to CLP. Mice were sacrificed and blood samples were collected 24 h after CLP. From the measured data, we can see that serum levels of IL-6 (96.76 ± 7.69 vs. 137.90 ± 7.44 pg/ml, *p* < 0.05), TNF-α (329.80 ± 16.52 vs. 401.90 ± 13.77 ng/L, *p* < 0.05), and cTnT (101.30 ± 2.46 vs. 109.70 ± 2.39 ng/L, *p* < 0.05) decreased (Figures 4A–C) and IL-10 (20.52 ± 0.83 vs. 14.01 ± 0.83 pg/ml, *p* < 0.05) increased (Figure 4D) in the rutin pretreatment group compared with the non-intervention CLP group.

### Rutin Alleviates Pathological Changes of Myocardial Inflammation in the CLP Mouse Model

**FIGURE 5:**
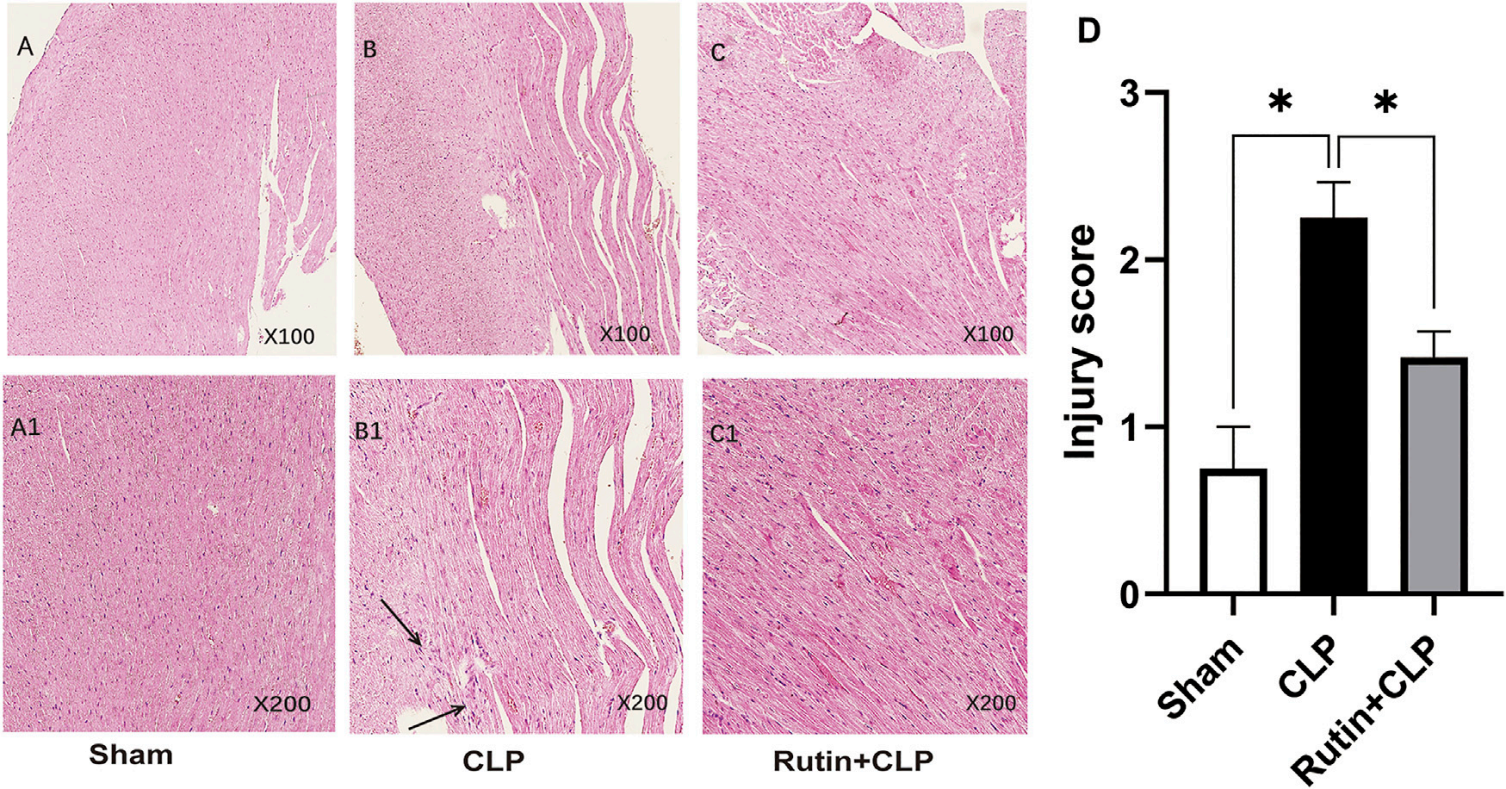
Rutin alleviates pathological changes of myocardial inflammation in the CLP mouse model. Histological images of H&E stained myocardial tissue slides stained with H&E. Male C57BL/6 mice were precautionary treated with rutin for 7 days and then subjected to CLP. Mice were sacrificed 24 h after surgery and cardiac tissue was obtained and stained with H&E. The arrow shows infiltration of inflammatory cells, interstitial edema, and myocardial fiber breakage. **(A,A1)**: sham group; **(B,B1)**: CLP group; **(C,C1)**: rutin+CLP group. **(D)** injury scores were assigned to H&E-stained myocardial tissue to show cardiac histology changes. Data represented the mean ± SEM of independent experiment in triplicate (*n* = 6 per group). Statistical significance (*p < 0.05) was analyzed using one-way ANOVA.

To determine whether pretreatment rutin attenuates cardiac inflammation, we used H&E staining to measure the histopathology of cardiac tissue. The results showed that the pathological changes of myocardial inflammation (Figures 5A–C1), such as inflammatory cell infiltration, interstitial edema, and myocardial fiber breakage, were significantly alleviated by pretreatment with rutin (2.250 ± 0.214 vs. 1.417 ± 0.154, *p* < 0.05) (Figure 5D).

### Rutin Pretreatment Improves Cardiac Function in CLP Induced Mouse Model

**FIGURE 6:**
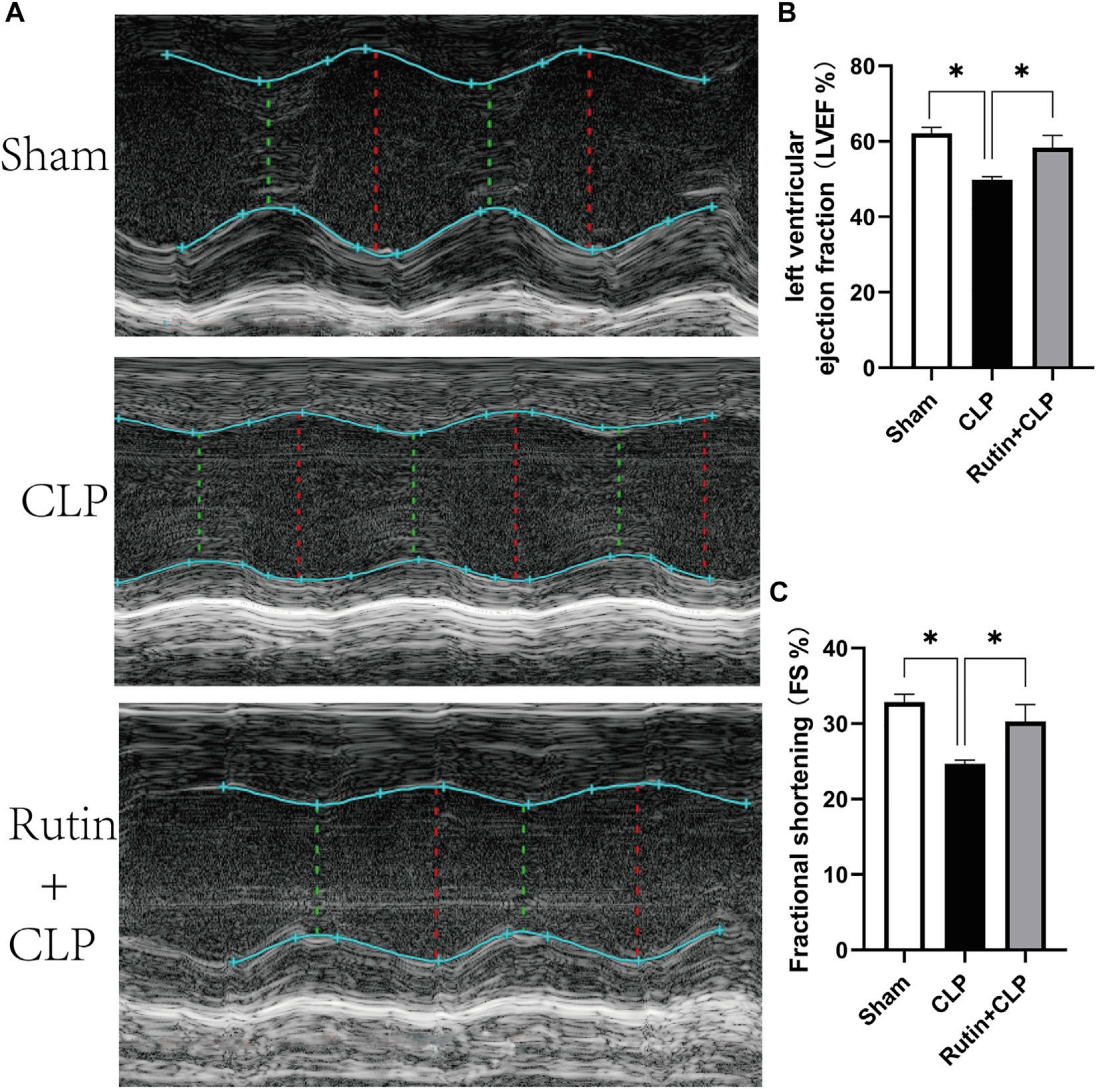
Rutin pretreatment improves cardiac function in CLP induced mouse model. Male C57BL/6 mice were precautionarily treated with rutin for 7 days, then subjected to CLP. 24 h later, cardiac function was measured by echocardiography. **(A)** Representative M-mode echocardiograms; Evaluation of cardiac function with LVEF **(B)** and FS **(C)**. Data represented the mean ± SEM of independent experiment in triplicate (*n* = 6 per group). Statistical significance (*p < 0.05) was analyzed using one-way ANOVA.

To investigate the protective effect of rutin on the cardiac function of the sepsis model, we use Two-dimensional and M-mode images to record left ventricular ejection fraction (LVEF) and left ventricular fractional shortening (FS). As shown in Figure 6A, pretreatment of rutin in CLP induced sepsis mouse markedly improved the LVEF (58.37 ± 3.21 vs. 49.82 ± 0.82, *p* < 0.05) and FS (30.28 ± 2.24 vs. 24.68 ± 0.46, *p* < 0.05), when compared with the non-intervention CLP group (Figures 6B,C).

### Rutin Reduces Cardiac Apoptosis in the CLP-Induced Sepsis Mouse Model

**FIGURE 7:**
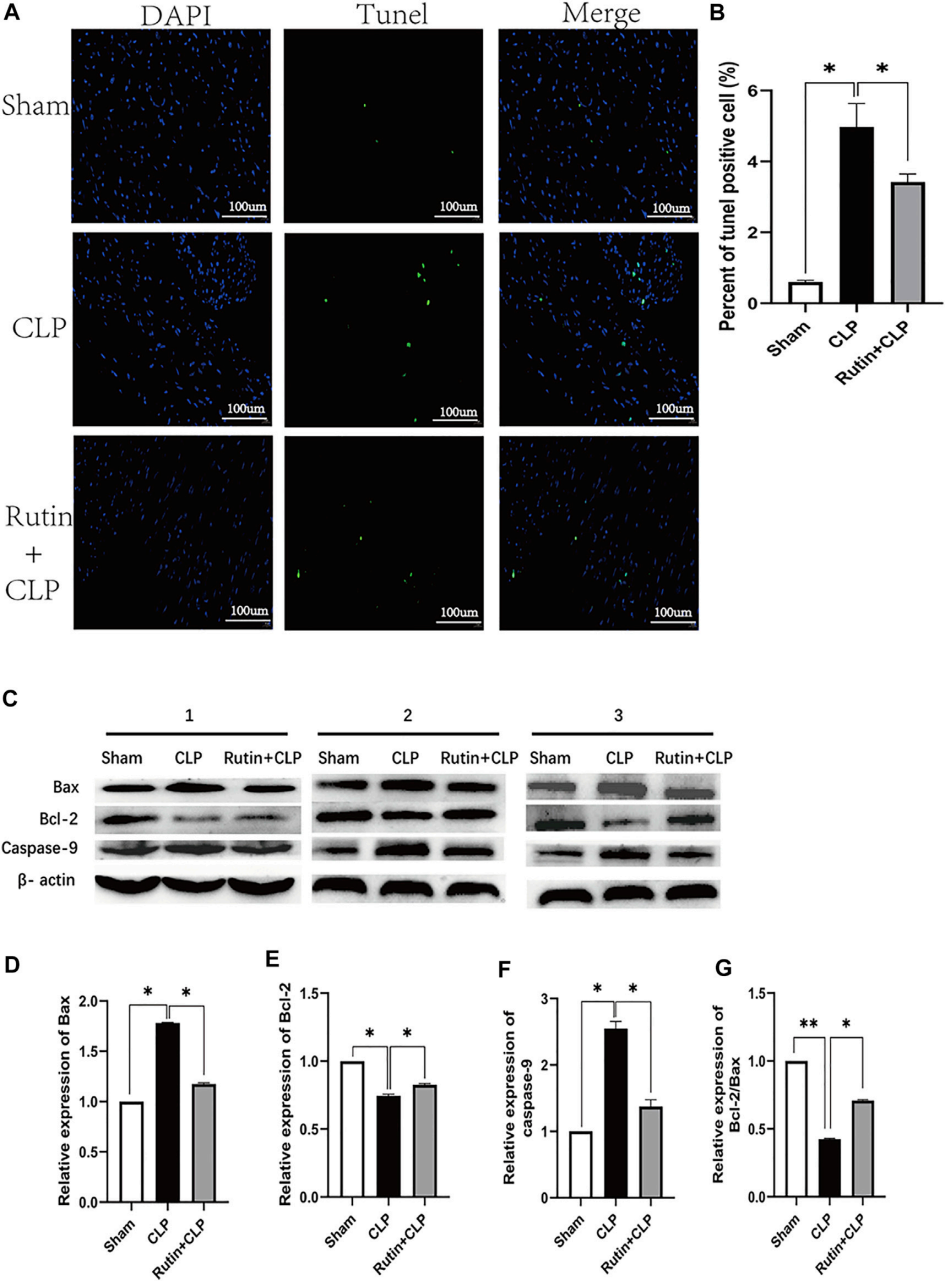
Rutin reduces cardiac apoptosis in CLP induced sepsis mouse model. Male C57BL/6 mice were precautionarily treated with rutin for 7 days, then subjected to CLP 24 h later mice were sacrificed, and the hearts were collected. **(A)** Representative TUNEL staining of apoptotic cardiomyocytes (green dots); Normal nuclei were stained with DAPI (blue dots). **(B)** Percentage of TUNEL positive cells in each group. **(C)** The expression of the Bax, Bcl-2, and Caspase-9 proteins in heart tissue was measured by Western blotting. Densitometric analysis of the expression of Bax, Bcl-2, and Caspase-9 **(D**–**F)** expression normalized to β-actin in each group. And Bcl-2/Bax **(G)** was also calculated and comparatively analyzed. Data represented the mean ± SEM of independent experiment in triplicate (*n* = 6 per group). Statistical significance (*p < 0.05) was analyzed using one-way ANOVA.

After demonstrating that the pretreatment of rutin improved cardiac function, we next investigated the effect of rutin on cardiac apoptosis in CLP mouse model (Figure 7). Compared to the sham group, the CLP group resulted in a significant increase in Bax levels and caspase-9 activation (Figure 7C). Compared with the non-intervention CLP group, the pretreatment of the rutin group, however, significantly attenuated the increases in Bax levels (1.179 ± 0.004 vs. 1.770 ± 0.012, *p* < 0.05) and the associated activation of caspase-9 (1.422 ± 0.061 vs. 2.590 ± 0.055, *p* < 0.05) (Figures 7D,E). Furthermore, CLP induced a decrease in Bcl-2 levels in the heart, which was also significantly alleviated by rutin pretreatment (0.837 ± 0.008 vs. 0.751 ± 0.008, *p* < 0.05) (Figure 7F). The effect of rutin on Bcl-2/Bax showed a similar trend to that of Bcl-2 (Figure 7G). We also performed TUNEL staining to confirm apoptosis in heart tissue. The CLP group resulted in an increase in relative TUNEL fluorescence compared to the sham group, and the CLP group with rutin pretreatment resulted in a decrease in relative TUNEL fluorescence compared to the non-treatment CLP group (3.421 ± 0.228 vs. 4.974 ± 0.664, *p* < 0.05) (Figures 7A,B).

### Effects of Rutin on the Mitochondria of Cardiomyocytes

**FIGURE 8:**
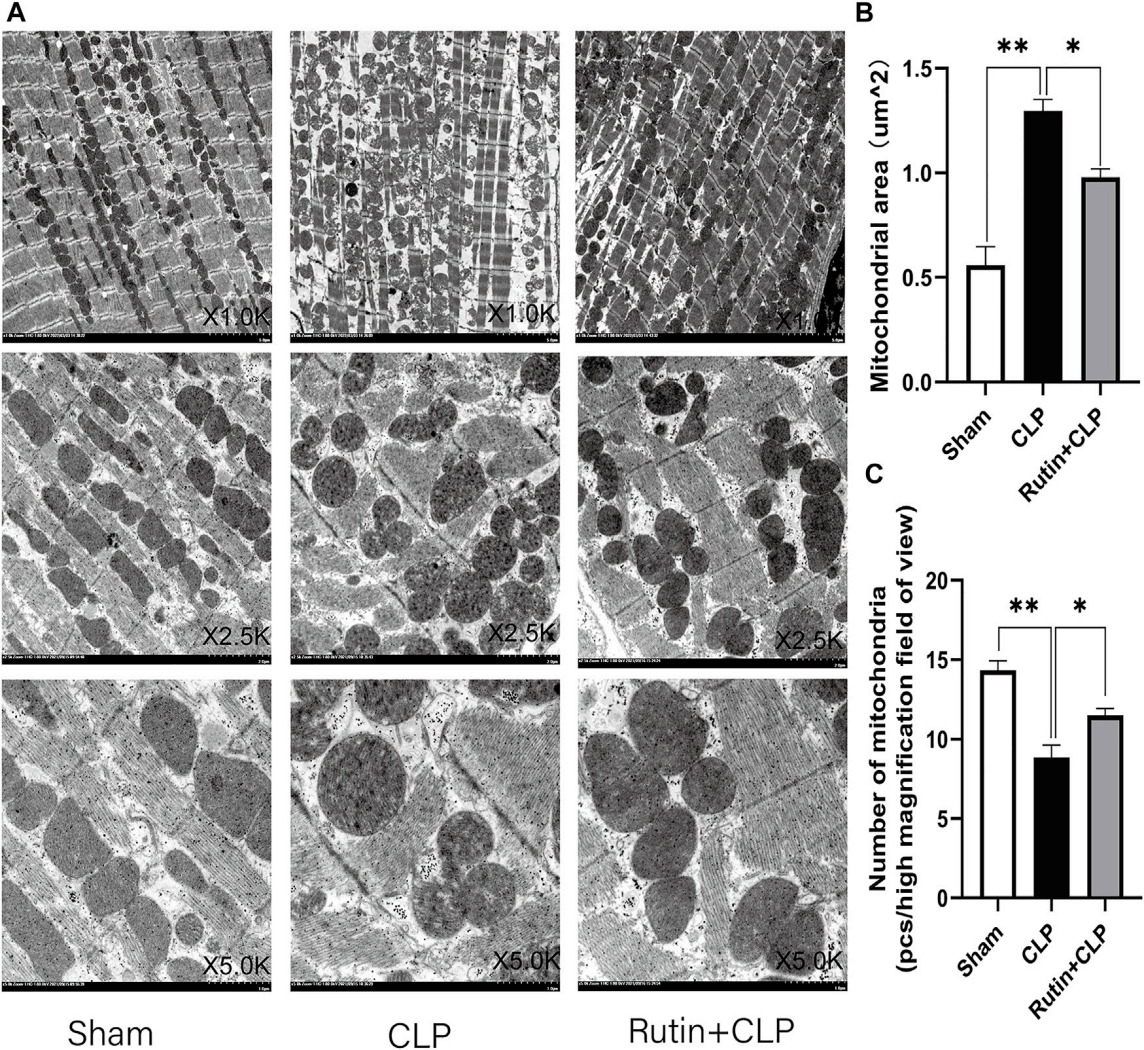
Effects of rutin on cardiomyocyte mitochondria. Myocardial tissue samples were freshly obtained 24 h after CLP, **(A)** transmission electron microscopy (TEM) was used to observe the mitochondria of cardiomyocytes. Image the same part with different magnifications. **(B,C)** Mitochondrial numbers and area were measured after 24 h of treatment. Data represented the mean ± SEM of independent experiment in triplicate (*n* = 6 per group). Statistical significance (*p < 0.05, **p < 0.01) was analyzed using one-way ANOVA.

To clarify the mechanism of rutin protection in cardiomyocytes, we observe the morphology of mitochondria in cardiomyocytes with transmission electron microscopy (TEM). The results showed that the number of myocardial mitochondria in the CLP group was reduced and there was swelling, vacuole-like changes and broken mitochondrial cristae compared to the sham group (Figure 8A). Furthermore, pretreatment with rutin in CLP mice significantly alleviated mitochondrial morphology change of mitochondria (1.009 ± 0.027 vs. 1.307 ± 0.0326, *p* < 0.05) and increased mitochondrial number of mitochondria (11.500 ± 0.428 vs. 8.833 ± 0.792, *p* < 0.05) (Figures 8B,C).

## Discussion

Rutin, with a protective effect of anti-inflammatory, antioxidant, antiallergic, and antiviral effects (Ganeshpurkar and Saluja, 2017b; Dudylina et al., 2019), has been widely used in the treatment of infections, cardiovascular diseases, diabetes, tumors, and other diseases (Xianchu et al., 2018; Ganesan et al., 2020; Lins et al., 2020). In our study, we found that pretreatment of rutin in a murine sepsis model protects the mouse from sepsis and SIC. The precautionarily treated rutin prevented SIC by inhibiting the apoptosis of cardiomyocytes and improving cardiac function. This protective effect is probably related to the restoration of the structure and function of myocardial mitochondria.

Sepsis is a lethal syndrome induced by infection (Hotchkiss et al., 2013), which can lead to multiple organ dysfunction and has an extremely high mortality rate. Complications with SIC will further increase mortality and hospitalization costs (Yende et al., 2016; Beesley et al., 2018). Treatment targeting the onset and development of septic cardiomyopathy can significantly improve the prognosis of sepsis (Røsjø et al., 2018; Bréchot et al., 2020; Hollenberg and Singer, 2021). Inflammatory factors, oxidative stress, and mitochondrial dysfunction play significant roles in the pathogenesis of SIC (Liu et al., 2017). Previous studies have shown that rutin reduces oxidative stress and alleviates myocardial damage in diabetic cardiomyopathy and coronary heart disease (Huang et al., 2017; Imam et al., 2017; Lv et al., 2018; Oluranti et al., 2021). In our study, prophylactic administration of rutin before the onset of sepsis could attenuate cardiomyocyte apoptosis and alleviate cardiac dysfunction in septic mice, which illustrated the protective effect of rutin on SIC.

Cardiomyocyte apoptosis is a key component of SIC (Cai et al., 2020; Qi et al., 2020), and inhibiting cardiomyocyte apoptosis can effectively improve myocardial injury in sepsis (Hong et al., 2021). Mitochondrial damage activates cardiomyocyte apoptosis, and pro-apoptotic proteins Bax and Bad translocate to the mitochondrial outer membrane and interact with it, causing the opening of the mitochondrial permeability transition pore (MPTP). The opening of the MPTP promotes cytochrome c released from mitochondria into the cytosol and combines with Apaf-1 to form a cytochrome C-Apaf-1 polymer. At the same time, Apaf-1 activates the original caspase-9 to form a cytochrome C/Apaf-1/caspase-9 complex known as the apoptosome. The apoptosome helps activate downstream caspase pathways such as caspase-3 and caspase-7 and exacerbate apoptosis (Xu et al., 2020; Zhou et al., 2021). Our study found that cardiomyocyte apoptosis increased in CLP-induced murine sepsis, and pretreatment with rutin significantly attenuated increases in Bax levels and associated activation of caspase-9 and reduced cardiomyocyte apoptosis in SIC.

Mitochondria act as the energy supplier of cardiomyocytes. When damaged, ATP synthesis will be blocked, causing cell damage (Larche et al., 2006). Swelling and vacuolation are signs of mitochondrial damage; swelling of the mitochondria is due to the activation of the mitochondrial apoptotic pathway, leading to apoptosis (Brentnall et al., 2013). The main cause of mitochondrial swelling is the increased permeability of the mitochondrial membrane, leading to the release of cytochrome c from mitochondria and activates factor-1 (Apaf-1)-dependent activation of procaspase-9 in the apoptosome, which initiates the external pathway of cardiomyocyte apoptosis (Brentnall et al., 2013). Qu et al. (2019) found that rutin can attenuate vancomycin-induced renal tubular cell apoptosis by inhibiting mitochondrial dysfunction. Here, we observed that precautionarily treated with rutin before the onset of sepsis alleviated SIC, and the changes in mitochondrial morphology and quantity was restored at the same time. We speculate that the protective effect of rutin on SIC may be related to the mitochondrial mechanism, which needs further verification in the future study.

This is the first time we have verified the protective effect of rutin in an animal sepsis model, providing a new direction for the prevention of sepsis. This study had limitations. First, this study did not verify the time-effect relationship of rutin and SIC. However, the time effect used in this study were all based on previous research (Xianchu et al., 2018). Second, there is no validation in clinical patients with sepsis. Third, we only observed changes in mitochondrial morphology and quantity, but not mitochondrial function. A causal relationship between the protective effects of rutin and mitochondrial mechanisms has not been demonstrated. Finally, all our studies were given prophylactic rutin before the onset of sepsis to observe the effect of rutin-enriched diet on the prevention of SIC. We did not verify the therapeutic effect of rutin on SIC after the onset of sepsis. These will be explored in-depth in our future research.

## Conclusion

In conclusion, our research confirmed the efficacy of rutin-enriched diet in the prophylaxis of cardiac apoptosis and cardiac injury induced by CLP in mouse model. It provides a potential new approach on SIC prophylaxis in sepsis.

## Data Availability

The original contributions presented in the study are included in the article/Supplementary Material, further inquiries can be directed to the corresponding authors.
